# Access to CD4 Testing for Rural HIV Patients: Findings from a Cohort Study in Zimbabwe

**DOI:** 10.1371/journal.pone.0129166

**Published:** 2015-06-17

**Authors:** Florian Vogt, Katie Tayler-Smith, Andrea Bernasconi, Eliphas Makondo, Fabian Taziwa, Buhlebenkosi Moyo, Liberty Havazvidi, Srinath Satyanarayana, Marcel Manzi, Mohammed Khogali, Anthony Reid

**Affiliations:** 1 Operational Centre Barcelona, Médecins sans Frontières/Doctors without Borders, Barcelona, Spain; 2 Operational Research Unit Luxembourg, Médecins sans Frontières/Doctors without Borders, Luxembourg, Luxembourg; 3 Department of Field Epidemiology and Training, Epicentre, Paris, France; 4 Laboratory Department, Beitbridge District Hospital, Ministry of Health and Child Welfare, Beitbridge, Zimbabwe; 5 Zimbabwe Mission, Médecins sans Frontières/Doctors without Borders, Harare, Zimbabwe; 6 Beitbridge Project, Médecins sans Frontières/Doctors without Borders, Beitbridge, Zimbabwe; 7 Centre for Operational Research, South-East Asia Regional Office, International Union against Tuberculosis and Lung Disease, New Delhi, India; Alberta Provincial Laboratory for Public Health/ University of Alberta, CANADA

## Abstract

**Background:**

CD4 cell count measurement remains an important diagnostic tool for HIV care in developing countries. Insufficient laboratory capacity in rural Sub-Saharan Africa is frequently mentioned but data on the impact at an individual patient level are lacking. Urban-rural discrepancies in CD4 testing have not been quantified to date. Such evidence is crucial for public health planning and to justify new yet more expensive diagnostic procedures that could circumvent access constraints in rural areas.

**Objective:**

To compare CD4 testing among rural and urban HIV patients during the first year of treatment.

**Methods:**

Records from 2,145 HIV positive adult patients from a Médecins sans Frontières (Doctors without Borders) HIV project in Beitbridge, Zimbabwe, during 2011 and 2012 were used for a retrospective cohort analysis. Covariate-adjusted risk ratios were calculated to estimate the effects of area of residence on CD4 testing at treatment initiation, six and 12 months among rural and urban patients.

**Findings:**

While the proportion of HIV patients returning for medical consultations at six and 12 months decreased at a similar rate in both patient groups, CD4 testing during consultations dropped to 21% and 8% for urban, and 2% and 1% for rural patients at six and 12 months, respectively. Risk ratios for missing CD4 testing were 0.8 (95% CI 0.7-0.9), 9.2 (95% CI 5.5-15.3), and 7.6 (95% 3.7-17.1) comparing rural versus urban patients at treatment initiation, six and 12 months, respectively.

**Conclusions:**

CD4 testing was low overall, and particularly poor in rural patients. Difficulties with specimen transportation were probably a major factor underlying this difference and requires new diagnostic approaches. Our findings point to severe health system constraints in providing CD4 testing overall that need to be addressed if effective monitoring of HIV patients is to be achieved, whether by alternative CD4 diagnostics or newly-recommended routine viral load testing.

## Introduction

Measurement of absolute cluster of differentiation type 4 (CD4+) cells/μL blood levels is important for initiating and monitoring anti-retroviral therapy (ART) in Human Immunodeficiency Virus (HIV) infected people. WHO guidelines recommend CD4 testing at ART initiation and at six month intervals in settings where viral load measurement is not available [1]. Zimbabwe is a resource-limited country in Sub-Saharan Africa (SSA) with an HIV prevalence of 15% among the adult population [2]. In line with WHO recommendations, national HIV guidelines between 2010 and 2013 recommended ART initiation for all HIV positive adults with CD4 levels below 350 cells/μL, or with WHO clinical stages three or four [3]. In mid-2013, this threshold was raised to 500 CD4 cells//μL, and viral load testing is now encouraged by WHO, if possible [4]. Wide scale implementation of these new recommendations in countries like Zimbabwe, however, is limited by financial constraints. In Zimbabwe, national guidelines thus recommend prioritizing patients with CD4 levels below 350 cells/μL and continue to recommend CD4 testing as the main diagnostic method for treatment initiation and monitoring [4]. For the initiation of preventive treatment of HIV coinfections such as toxoplasmosis or cryptoccocal meningitis, CD4 testing remains the method of choice to define treatment eligibility according to WHO recommendations [5,6].

In Zimbabwe, as in other SSA countries, CD4 testing capacity has not kept up with the continuous ART decentralization process from urban to peripheral health facilities [7]. This is due to high costs of diagnostic equipment, poor facility infrastructure and lack of skilled staff [8,9]. As a result, CD4 diagnostics remain limited to central laboratories in urban settings, which requires sample transportation between rural and central facilities [7,10,11]. As in most SSA countries, Ethylenediaminetetraacetic Acid (EDTA) tubes are widely used in Zimbabwe for CD4 blood collection. Samples collected in EDTA tubes must be analysed within 24 hours, requiring same-day transportation and processing from rural to central facilities. This, however, is often not possible, particularly in remote areas or during the rainy season.

The effects of these constraints on access to CD4 testing for patients from rural areas in SSA remain poorly understood. The challenges of providing HIV diagnostics and care in rural SSA are frequently reported in the literature, but most often without explicitly comparing rural versus urban areas [12]. Studies that do assess HIV diagnostic issues lack a quantification of the impact at an individual patient level [11,13], and tend to focus on patient outcomes [9,14,15], ART coverage [16–19], or voluntary testing and counselling [9,20–22]. CD4 testing in rural patients for treatment initiation and monitoring is often overlooked, or where it is assessed, explicit quantification of the difference between rural and urban patients is lacking [8,10,14,23]. To our knowledge, there are no studies that compare and quantify differences in WHO recommended CD4 testing practices between HIV patients from rural and urban areas during the first year on treatment.

Evidence regarding the existence and magnitude of differences in access to CD4 testing in disadvantaged populations is crucial for public health planning and to support changes in existing service delivery. For instance, alternative diagnostic procedures that could circumvent sample transport constraints such as use of dried blood spots, blood fixatives, point-of-care CD4 enumeration, or CD4 Stabilization Tubes are often associated with higher costs and hence not affordable for many national AIDS programmes. Their introduction would require sound justification. In addition, if routine viral load testing is to become a reality as a treatment monitoring tool in Zimbabwe and other SSA countries soon, it will likely require overcoming similar challenges in specimen transport [10]. Hence the need for more detailed evidence on the differences in access.

In a Médecins sans Frontières / Doctors Without Borders (MSF) HIV project in Beitbridge district, Zimbabwe, we compared access to CD4 testing among HIV patients from rural and urban areas at ART initiation, at six and at 12 months of treatment in a retrospective cohort analysis.

## Material and Methods

### Setting

This study was conducted in Beitbridge district, Matabeleland South province, where MSF maintained an HIV project supporting Ministry of Health and Child Welfare (MoHCW) in prevention, treatment and care of HIV/AIDS between 2009 and 2013. Beitbridge district is located in the south of Zimbabwe bordering South Africa with an estimated population of about 122,000 of which about 34% live in rural areas [24]. HIV prevalence in Matabeleland South province, where Beitbridge district is located, is 21.2%, which is substantially higher than the national average of 15.2% [2]. Its population is very mobile, with a high volume of legal and illegal cross-border movements to South Africa for commercial and farming purposes. This further compromises access to health care, in particular for chronic conditions such as HIV/AIDS. Irregular uptake of medication is common and loss-to-follow-up from care is high.

In close collaboration with MoHCW, MSF offered comprehensive ART services at Beitbridge District Hospital (BDH) and at six rural health centres (RHC) free of charge across the district between 2009 and 2013. BDH laboratory was the only public facility in the district providing comprehensive HIV diagnostic services, including absolute CD4 count measurement using BD Facscount (Becton Dickinson, Franklin Lakes, New Jersey, USA) and Partec Cyflow (Partec GmbH, Muenster, Germany) cytometers. Collection of blood samples was done using EDTA tubes. The road network in the district is patchy, distances between BDH and RHCs are long, and many roads become impassable during the rainy season.

A joint outreach team of MSF and MoHCW nurses regularly visited one RHC per day on an alternating basis to provide HIV care. RHCs were visited once per week on average. At the end of outreach days, patient samples were brought back to BDH for laboratory analysis by the outreach team. Sample transportation on days not coinciding with outreach team visits was erratic due to the limited transport capacity of MoHCW. As such, CD4 testing at RHCs was usually restricted to days when an MSF/MHCW outreach team visited the facility.

At BDH and RHCs, medical information of patients was recorded on paper-based patient files during consultations by MoHCW nurses. On an ongoing basis throughout the project, the standard MSF electronic database “Follow Up Care of HIV/Aids” (FUCHIA) [25] was maintained based on the MoHCW patient files. All patients starting ART in the project were registered in FUCHIA, and information was updated by MSF data encoders after each patient visit. Data cleaning and validation was done by the MSF data manager in a standardized way on a concurrent basis.

### Study population

For this analysis, all records from HIV positive patients above 18 years of age initiated on ART at BDH or any of the six supported RHCs in the MSF HIV project in Beitbridge between January 2011 and December 2012 were considered eligible.

### Data

Records were sourced from the FUCHIA database and the following variables were included for this analysis: area of residence (patients residing in the BDH catchment area were defined as “urban” and patients residing in the catchment area of one of the RHCs as “rural”), whether or not a CD4 test result was recorded at ART initiation, at six and at 12 months (+/-30 days), and baseline characteristics at enrolment (WHO clinical stage, sex, age, profession and marital status). See S1 Table.

### Analysis

The exposure of interest was area of residence (urban/rural), and the outcome of interest, whether a patient had a CD4 test result recorded at the different time points. Socio-demographic and clinical patient characteristics were treated as covariates. A bivariate analysis based on chi-square tests was performed to compare the distribution of baseline characteristics between both patient groups. Crude and covariate-adjusted risk ratios including 95% confidence intervals and p-values were calculated using binomial regression models to compare the association between area of residence and whether a CD4 test was done at ART initiation, six and 12 months. All baseline covariates were considered as *a priori* confounders and included in the adjusted model. Type 1 error margins of 5% were applied throughout. Analysis was conducted between July 2013 and June 2014 using software package STATA v.11 [26].

### Ethics Statement

This study is based on existing data from routine project activities that had already been collected under a Memorandum of Understanding between MoHCW and MSF for monitoring and evaluation purposes. Data were analyzed anonymously and only aggregated data without personally identifiable information is presented, hence no individual informed consent was required. Exemption from ethics review was granted by the Medical Research Council of Zimbabwe. This study met the Médecins Sans Frontières’ (Geneva, Switzerland) Ethics Review Board-approved criteria for analysis of routinely-collected programme data. It also satisfied the requirements of the Ethics Advisory Group of the International Union against Tuberculosis and Lung Disease (Paris, France) and meets their approval. This study was conducted in accordance with the principales of the Helsinki Declaration.

## Results

There were 2,222 records that matched the inclusion criteria. Of these, 77 (3%) were excluded (34 duplicate records; 43 records with missing data), leaving records of 2,145 patients for the analysis. Patient baseline characteristics are described in Table 1.

**Table 1:** Characteristics of rural vs urban HIV patients in Beitbridge, Zimbabwe, at ART initiation between January 2011 and December 2012.

	Rural		Urban		
	N = 600		N = 1,545		
	n	%	n	%	P-value*
**Sex**					0.022
Male	193	(32)	586	(38)	
Female	394	(66)	947	(61)	
*missing*	*13*	*(2)*	*12*	*(<1)*	
**Age** *(years)*					<0.001
18–30	137	(23)	460	(30)	
31–40	182	(30)	586	(38)	
>40	281	(47)	499	(32)	
**Profession**					<0.001
Employed	86	(14)	431	(28)	
Unemployed	409	(68)	849	(55)	
Other	105	(18)	258	(17)	
*missing*	*0*	*(0)*	*7*	*(<1)*	
**Marital status**					<0.001
Single	63	(10)	316	(21)	
Married	330	(55)	846	(55)	
Other	185	(31)	360	(23)	
*missing*	*22*	*(4)*	*23*	*(1)*	
**WHO clinical stage**					<0.001
1	23	(4)	99	(6)	
2	229	(38)	331	(21)	
3	325	(54)	1036	(67)	
4	23	(4)	78	(5)	
*missing*	*0*	*(0)*	*1*	*(<1)*	

HIV: Human Immuno-Deficiency Virus, ART: Anti-Retroviral Therapy, WHO: World Health Organization, N: Total, n: Subtotal

* Chi-Square test

Adjusting for socio-demographic and clinical differences between patient groups, the risks for missing CD4 testing were 0.8 (95% CI 0.7–0.9) times lower, 9.2 (95% CI 5.5–15.3) times higher and 7.6 (95% 3.7–17.1) times higher for rural patients compared to urban patients at ART initiation, six months and twelve months, respectively. The inclusion of covariates had no substantial effect on the association between area of residence and CD4 testing (Table 2).

**Table 2:** Association between rural vs urban residence and CD4 testing among HIV patients in Beitbridge, Zimbabwe, initiated on ART between January 2011 and December 2012.

	Patients attending consultations	Patients not receiving CD4 test result**		Crude RR	Adjusted RR***	95% CI	P-value****
		n	%				
At ART initiation*	N = 2,145						
Urban	1,545	886	(57)	1	1		
Rural	600	283	(47)	0.8	0.8	(0.7–0.9)	<0.001
At 6 months*	N = 1,250						
Urban	889	704	(79)	1	1		
Rural	361	352	(98)	9.7	9.2	(5.5–15.3)	<0.001
At 12 months*	N = 1,199						
Urban	845	774	(92)	1	1		
Rural	354	350	(99)	8.0	7.6	(3.7–17.1)	<0.001

CD4: Cluster Differentiation Type 4, HIV: Human Immuno-Deficiency Virus, ART: Anti-Retroviral Therapy, WHO: World Health Organization, RR: Relative Risk, CI: Confidence Interval, N: Total. n: Subtotal

* +/-30 days; separately for each time point irrespective of care received previously

** During consultations as recommended by WHO

*** Adjusted for: age, sex, profession, marital status, WHO status

**** Likelihood-Ratio test

**Fig 1:**
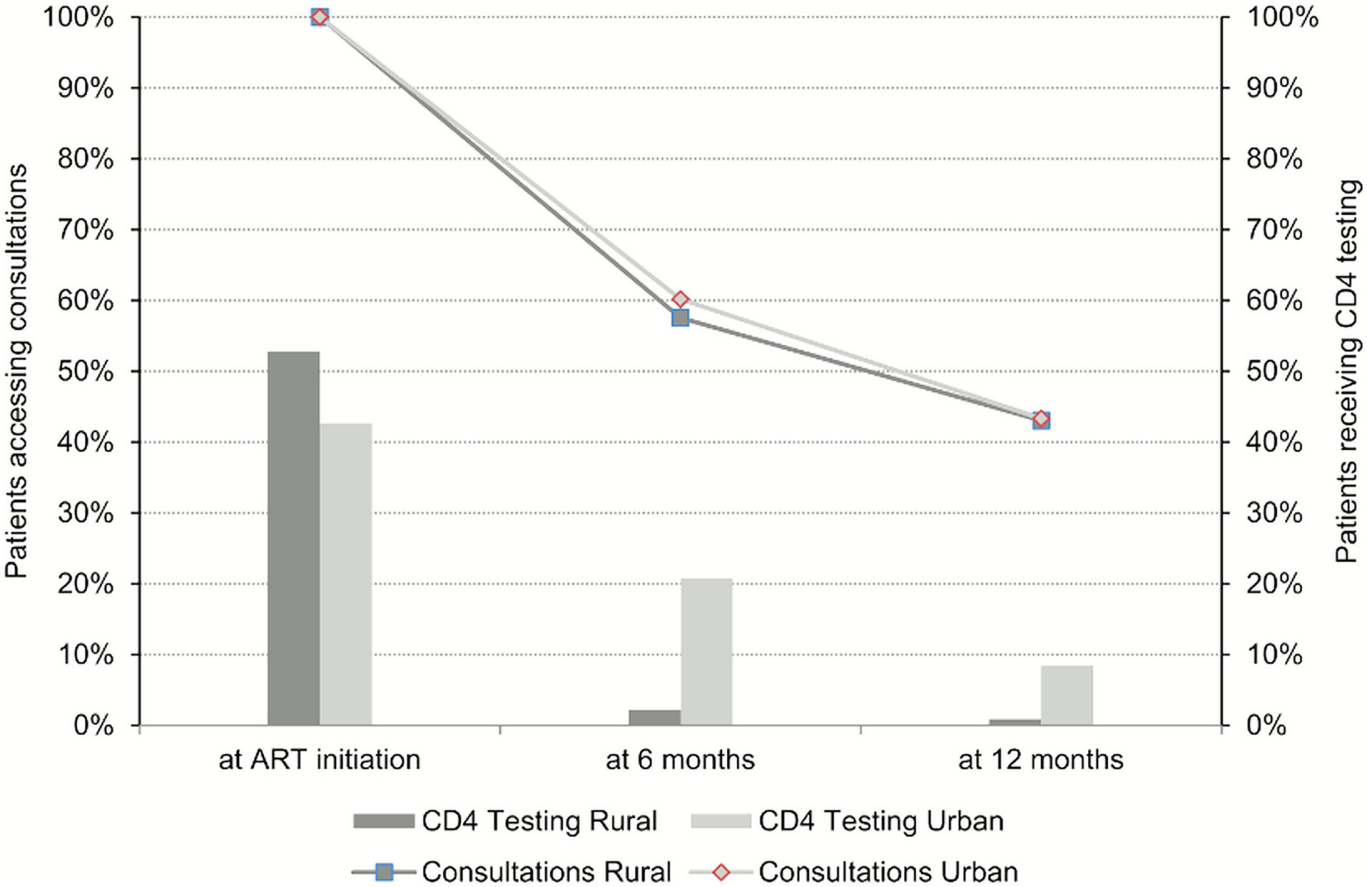
Proportions of rural vs urban patients accessing consultations, and proportions receiving CD4 testing during consultations, during first year of anti-retroviral therapy in Beitbridge, Zimbabwe, between January 2011 and December 2012.

While the proportion of patients accessing medical consultations decreased at six and 12 months of treatment progressively, the rate of decline in visits was similar in rural and urban patients (Fig 1). Of those patients coming for consultations, the proportion with CD4 results recorded at six and at 12 months (+/-30 days) dropped precipitously in both groups, particularly in rural patients at six and 12 months. At these two visits, almost no patients from rural areas had a CD4 result recorded (Fig 1).

Among all patients initiated on ART, the proportion of patients that received all CD4 tests as recommended during the course of their first year on treatment decreased from 43% and 53% at initiation to 15% and 2% at six months, and to 1% and 0% at 12 months, for urban and rural patients, respectively.

## Discussion

This is the first study to compare and quantify differences in CD4 testing between HIV patients from rural and urban areas in a SSA setting during the first year of ART. While our findings show that rural patients among the studied population are much less likely to have CD4 testing done when they should, according to WHO recommendations, at six and 12 months compared to urban patients, we found startlingly low CD4 testing in both patient groups.

The main strengths of this study are that i) a large number of patients, enrolled over two years, were included and followed up during their first year of treatment; ii) data were sourced from a standardized and internationally used database; iii) given the low number of excluded records, we are confident that our study sample is representative of the MSF HIV cohort initiated in Beitbridge in 2011 and 2012.

A number of limitations are recognized. First, our study only included patients from the six MSF-supported RHCs out of 13 RHCs districtwide, plus BDH. Treatment coverage among ART eligible HIV patients in Beitbridge district was about 35% (MSF project monitoring tool (logframe) 2013). While MSF chose to support RHCs based on remoteness and the intention to have fair geographical coverage, we cannot presuppose representativeness of our cohort for the the district or Zimbabwe as a whole. In fact, we may even underestimate the disparity in CD4 testing between urban and rural areas since MSF offered reliable and comprehensive care at RHCs during outreach days, which might have stimulated uptake of health services in these areas.

Second, data were collected for routine reporting purposes and we were unable to cross-check our study data with patient records for this analysis; nonetheless, data are believed to be robust due to built-in data entry checks in FUCHIA.

Third, although we controlled for basic demographic and clinical differences between patient groups, we cannot rule out residual confounding by other factors such as socio-economic status, health seeking behavior, number of patient clinic visits, or differences in service provision between urban and rural health facilities. In addition, mobility might have been different, being potentially more cyclical in rural areas depending on farming seasons and more constant in urban areas for commercial activities.

Our findings raise a number of important points for discussion:

Until now, the disadvantaged situation in rural areas in SSA with regards to CD4 testing had only been explored from a health system or laboratory infrastructure perspective. It had not yet been quantified at a patient level [11,13]. Our findings from Zimbabwe reveal that rural patients have substantially lower access to CD4 testing at time points recommended by WHO under the current system with a surprisingly large effect size. This provides much-needed evidence to lobby for changes in health care delivery at national and international level even if it requires financial investment in new diagnostic technologies such as use of dried blood spots or point-of-care devices [13]

By differentiating between consultations accessed and test results received, we were able to suggest that the reasons for poorer access to CD4 testing in rural compared to urban areas are likely to be within the health care system rather than with the patients. The current EDTA tube based testing method relies on timely specimen transportation and as such, given the transport problems during days when no MSF/MoHCW outreach visits were done, probably explains the bulk of the rural-urban discrepancy. Other explanations could be human resources constraints or lack of phlebotomy equipment. However, since MSF was supplying materials to RHCs during stock-out periods, we think that the latter factors are unlikely to explain much of the observed rural-urban discrepancy. The observed higher proportion of patients receiving CD4 testing at baseline in rural compared to urban areas is striking. A possible explanation for this finding may be related to differences in levels of training between rural and urban health care workers. Rural health care workers often have a lower level of training compared to urban staff, and as such might place greater reliance on the certainty of CD4 testing, instead of clinical assessment, to initiate ART compared to health staff in urban areas. This would lead to a relative increase the proportion of patients initiated on ART who receive CD4 testing at baseline in rural areas.

Overall, proportions of being CD4 tested were very low in urban as well as in rural patients, suggesting severe underlying health system constraints in the provision of timely CD4 testing that go beyond transport issues. Parts of the gap might be due to CD4 tests being done outside the precision window of +/-30 days. Patients tested outside this time period who returned within the time window shortly thereafter might not have been re-tested during that subsequent consultation. This implies rational clinical practice, but might have led to an overestimation of the problem in our study. However, given the magnitude of the observed gaps, it is unlikely that this fully explains the extent of our findings. Data capture of CD4 testing into the FUCHIA database was done from returned laboratory result slips in patient files and as such we could not determine where between patient contact and filing of result slip the processing chain broke down. Possible reasons for patients in both areas not receiving effective CD4 testing when they should have (according to WHO recommendations) may have included: patients not being bled during consultations; specimen handling issues in the laboratory; errors in returning of result slips into patient files; stock ruptures of cytometer consumables in the national supply system; or intermittent cytometer breakdowns.

The setting of this study—one of the districts in Zimbabwe with the highest HIV prevalence [2]—has a rural-urban pattern comparable to other districts in the country, and the public health care system suffers from financial, human resources and logistics constraints that are similar to other resource-limited high HIV prevalence countries. However, due to Zimbabwe´s unique political and economic situation, our findings should only be extrapolated to other settings in SSA with utmost caution.

To improve CD4 testing for rural patients, either sample transportation needs to be drastically improved or alternatives to the current testing methods need to be considered [27]. Point-of-care enumeration is a promising alternative to currently-used EDTA tubes [28,29] and is increasingly being implemented in rural SSA settings [30–33]. However, there have been mixed results from its performance in operational settings in SSA [34,35]. The use of CD4 Stabilization Tubes (Becton Dickinson, Franklin Lakes, New Jersey, USA) circumvent the need for same-day sample analysis, but this approach requires more evaluation in operational settings [36,37]. Viral load enumeration is now being encouraged as the cornerstone of treatment monitoring [38]. If it becomes affordable for widespread use in SSA soon, it should be done based on dried blood spot (DBS) techniques in rural areas, as any system that relies on time-restricted specimen transportation is unlikely to be successful in such settings [28,39]. Dried blood spot based viral load enumeration has been successfully tested in SSA settings but still relies on a well-functioning health system during routine use [38,40,41]. Ideally, a viral load Point-of-care test providing immediate results would be needed.

## Conclusions

In conclusion, this study found low access to CD4 testing at time points recommended by WHO (+/- 30 days) in a high HIV burden district in Zimbabwe. Rural patients were particularly disadvantaged, most likely due to difficulties in specimen transportation, and might benefit from the use of new context-adapted diagnostic methods. However, any innovation will have limited overall impact on the majority of patients without a well-managed health system to support it. Hasty roll out of universal viral load monitoring may face a similar fate as current CD4 testing methods.

## Supporting Information

S1 Table Study database MSF Beitbridge Project. (ZIP)
